# Beam polarization effects on top-pair production at the ILC

**DOI:** 10.1140/epjc/s10052-018-5895-9

**Published:** 2018-05-28

**Authors:** Nhi M. U. Quach, Yoshimasa Kurihara, Khiem H. Phan, Takahiro Ueda

**Affiliations:** 1 The Graduate University for Advanced Studies (SOKENDAI), Hayama, Kanagawa 240-0193 Japan; 2 High Energy Accelerator Research Organization (KEK), Tsukuba, Ibaraki 305-0801 Japan; 3 University of Science Ho Chi Minh City, 227 Nguyen Van Cu, Dist. 5, Ho Chi Minh City, Vietnam; 4 Nikhef, Science Park 105, 1098 XG Amsterdam, The Netherlands

## Abstract

Full one-loop electroweak corrections for an $e^-e^+\rightarrow t \bar{t}$ process associated with sequential $t\rightarrow b \mu \nu _\mu$ decay are discussed. At the one-loop level, the spin-polarization effects of the initial electron and positron beams are included in the total and differential cross sections. A narrow-width approximation is used to treat the top-quark production and decay while including full spin correlations between them. We observed that the radiative corrections due to the weak interaction have a large polarization dependence on both the total and the differential cross sections. Therefore, experimental observables that depend on angular distributions such as the forward–backward asymmetry of the top-production angle must be treated carefully including radiative corrections. We also observed that the energy distribution of bottom quarks is largely affected by the radiative corrections.

## Introduction

The discovery of the Higgs boson [1, 2] in 2012 showed the standard theory of particle physics to be well established. Even though the standard theory can describe the microscopic nature at a subatomic level very precisely [3], it cannot be the most fundamental theory of nature because, for instance, it includes many parameters (e.g., particle masses and couplings, number of generations) that are not determined within the theory. While experiments at the Large Hadron Collider continue to search for signals beyond the standard model (BSM), none have been reported to date.^1^ Besides discovering new particles, pursuing the BSM also involves precise measurements of the properties of known particles. Milestones along this direction must surely be the Higgs boson and the top quark. Because the top quark is the heaviest fermion with a mass above even the electroweak symmetry-breaking scale, it is naturally expected to play a special role in the BSM. In addition, it has been pointed out that the vacuum stability of the Higgs potential depends strongly on the Higgs and top-quark masses [6]. Hence, the precise measurement of top-quark properties is crucial for understanding the stability of the universe, as well as for the search for BSM signals.

The International Linear Collider (ILC) [7], which is a proposed electron–positron colliding experiment with center-of-mass (CM) energies above 250 GeV, is being discussed intensively as a future project in high-energy physics. The main goals of ILC experiments would be a precise measurement of the Higgs and top-quark properties and searching directly for new particles. The ILC will use spin-polarized beams for both electron and positron beams [8, 9] to increase its sensitivity to new physics and to improve its measurement accuracy. The design values of the beam polarization are 80% for the electron beam and $30\%$ for the positron beam with beam energies below 1000 GeV [10]. For many processes, beam polarization is a simple way to increase the signal cross section while suppressing the background. Moreover, beam polarization allows new properties to be measure (e.g., the polarization dependence of cross sections). Detailed Monte Carlo studies have shown that the ILC would be able to measure most of the standard model parameters to within sub-percent levels [11].

Because of the improved experimental accuracy intended of the ILC, theoretical predictions must be given with new level of precision. In particular, a radiative correction due to the electroweak interaction (including spin polarizations) is mandatory for such requirements. Before the discovery of the top quark, a full electroweak radiative correction was conducted for an $e^-e^+\rightarrow t\bar{t}$ process at a lower energy [12], and it was then obtained independently for higher energies [13, 14]. The same correction including radiative photon, the $e^-e^+\rightarrow t\bar{t}\gamma$ process, has also been reported [15]. Higher-order corrections including photon radiation are important for the precise prediction of cross sections because the initial photon radiation affects the total cross sections significantly. However, none of previous calculations include the effect of spin polarization. Some application of polarized cross sections of this process including full $\mathcal {O}(\alpha )$ electroweak corrections is reported in Ref. [16], in which polarized cross sections are obtained using the method presented here by the authors of the current report.

In the present study, we report full electroweak radiative corrections for the process $e^-e^+\rightarrow t \bar{t}\rightarrow b \bar{b} \mu ^+\mu ^-\nu _{\mu } \bar{\nu }_{\mu }$ using a narrow-width approximation for the top quarks. Spin-polarization effects are included, not only in the initial beams, but also in the full spin correlations of the production and decay of top quarks. While Born cross sections of the process $e^-e^+\rightarrow b \bar{b} \mu ^+\mu ^-\nu _{\mu } \bar{\nu }_{\mu }$ including all six-body final state are given in Ref. [17], an electroweak radiative correction of the $t\bar{t}$ process associated by their decay including a spin correlation is not calculated yet. On the other hand, NLO QCD corrections for on-shell $t\bar{t}$ and $t\bar{t}H$ including decays are calculated in Ref. [18]. A detailed study of the electroweak correction on the top-quark decay is also reported in Refs. [19, 20].

This report is organized as follows. The calculation method is explained in Sect. 2. We use the GRACE-Loop system to calculate the cross sections. A system-checking method is also explained in Sect. 2. In Sect. 3, we show results of electroweak corrections of the total cross section as well as of the angular distribution with spin-polarized beams. The effects of radiative corrections on top-quark decay products, including a spin correlation, are also discussed using a narrow-width approximation. The contribution of an NLO–QCD correction is briefly discussed in Sect. 3. We summarize and conclude this report in Sect. 4. In Appendix A, we summarize the formulas of the NLO–QCD correction for massive quark production.

## Calculation method

For precise cross-section calculations of the target process in this study, we used the GRACE-Loop system, which is an automatic system for calculating cross sections of scattering processes at one-loop level for the standard theory [21] and the minimal supersymmetric standard model [22]. This system has been used to treat electroweak processes with two, three, or four particles in the final state [23–26]. The GRACE-Loop system has the following features: (1) The renormalization of the electroweak interaction is carried out using an on-shell scheme [27, 28]. (2) The infrared divergences are regulated using a fictitious photon mass $\lambda$ [28]. (3) The symbolic manipulation system FORM [29] is used to handle all Dirac and tensor algebras in *n* space-time dimensions. (4) GRACE generates FORTRAN source code that calls library subroutines to calculate the scattering amplitudes. (5) For loop integrations, all tensor one-loop integrals are reduced to scalar integrals using our own formalism, whereupon the integrations are performed using the packages FF [30] and LoopTools [31]. (6) Phase-space integrations are done using an adaptive Monte Carlo integration package BASES [32, 33]. (7) For numerical calculations, we use quadruple precision for floating-point variables.

To treat spin polarization in loop calculations, the projection operators are applied on fermion wave functions. A spin projection of the initial beams is realized simply by multiplying the spin-projection operator , where *p* is the four-momentum of beam particles and $\lambda =\pm 1$ is their helicity. Here, we assume that the initial beams comprise light fermions with no transverse momenta. The electron/positron completeness relation becomes . For top quarks, the spin-polarization vector can be taken as

$$\begin{aligned} s^{\mu }_t= & {} \left( \frac{\varvec{p}_t\cdot \hat{\varvec{s}}_t}{m_t}, \hat{\varvec{s}}_t+ \frac{ (\varvec{p}_t\cdot \hat{\varvec{s}}_t)\varvec{p}_t }{m_t(E_t+m_t)} \right) , \end{aligned}$$

where $m_t$ is the top-quark mass, $E_t$ is the top-quark energy, and $\varvec{p}_t$ is the top-quark three-momentum. The spin is projected on the direction of the top-quark three-momentum using a direction vector $\hat{\varvec{s}}_t=\varvec{p}_t/|\varvec{p}_t|$. The completeness relations in this case are given as  for top quarks and  for anti-top quarks.

In GRACE, while using the $R_\xi$-gauge in the linear gauge-fixing terms, the non-linear gauge-fixing Lagrangian [21, 34] is employed, namely

$$\begin{aligned} \mathcal {L}_{GF}= & {} -\,\frac{1}{\xi _W}\left| \left( \partial _{\mu }-ie {\varvec{\tilde{\alpha }}} A_{\mu }-igc_W\varvec{\tilde{\beta }} Z_{\mu }\right) W^{\mu +}\right. \\&\left. +\,\xi _W \frac{g}{2}\left( v+\varvec{\tilde{\delta }} H + i\varvec{\tilde{\kappa }}\chi _3\right) \chi ^+\right| ^2\\&-\,\frac{1}{2\xi _Z}\left( \partial \cdot Z + \xi _Z\frac{g}{2c_W} \left( v+\varvec{\tilde{\varepsilon }} H\right) \chi _3\right) ^2\\&-\,\frac{1}{2\xi _A}\left( \partial \cdot A\right) ^2, \end{aligned}$$

for the sake of system checking. Here $A,Z,W,\chi$, and *H* denote the wave functions of the corresponding fields, and $\xi$’s are gauge parameters for the linear gauge-fixing terms. The results must be independent of the non-linear gauge parameters $\{\varvec{\tilde{\alpha },\tilde{\beta },\tilde{\delta },\tilde{\kappa },\tilde{\varepsilon }}\}$. We can perform system checking numerically to confirm the correctness of the system. Before calculating cross sections, we checked for ultra-violet coefficient ($C_{\mathrm{UV}}$) independence, photon-mass ($\lambda$) independence, and gauge invariance numerically at several randomly chosen phase points. For instance, in the polarized case at a CM energy of 500 GeV, we confirmed ultra-violet coefficient and photon-mass independence, both with stable results over 19 digits, when the parameters $C_{\mathrm{UV}}$ and $\lambda$ changed by three orders of magnitude from their nominal values. Meanwhile, the non-linear gauge-invariance results are stable over 25 digits against changing those values. Even a light-fermion Yukawa coupling cannot be neglected to achieve such precision. We note that the parameter dependence of the amplitude is logarithmic for $C_{\mathrm{UV}}$ and $\lambda$, while it is up to quartic for the non-linear gauge parameters. In addition to the above checks, we examined the soft-photon cut-off independence: for cross sections at the one-loop level, the results must be independent of a hard-photon cut-off parameter $k_c$. We confirmed that the integration results are self-consistent within the statistical error of numerical phase-space integrations while varying $k_c$ from $10^{-4}$ to $10^{-1}$ GeV.

**Table 1:** Input parameters

*u*-quark mass	$58.0\times 10^{-3}$ GeV	*d*-quark mass	$58.0\times 10^{-3}$ GeV
*c*-quark mass	1.5 GeV	*s*-quark mass	$92.0\times 10^{-3}$ GeV
*t*-quark mass	173.5 GeV	*b*-quark mass	4.7 GeV
*Z*-boson mass	91.187 GeV	*W*-boson mass	80.370 GeV
*Z*-boson width	2.356 GeV	*W*-boson width	1.993 GeV
Higgs mass	126 GeV		

## Results and discussions

For cross-section calculations of the production process $e^-e^+ \rightarrow t \bar{t}$ and its sequential top decay, we use the input parameters listed in Table 1. The masses of the light quarks (i.e., other than the top quark) and *W* boson are chosen to be consistent with low-energy experiments [35]. Other particle masses (including an electron mass) are taken from recent measurements [3]. For renormalization scheme, the on-shell scheme is used, in which input parameters to determine electroweak couplings are *W*-boson and *Z*-boson masses, and the fine-structure constant. The Weinberg mixing angle is obtained using the on-shell condition, $\sin ^2{\theta _W} = 1- m_W^2/m_Z^2$. The fine-structure constant $\alpha =1/137.0359859$ is taken from the low-energy limit of Thomson scattering due to our renormalization scheme. The *W*-boson and *Z*-boson widths are taken as the calculated value at tree level using the same parameters given above.

### Production cross sections

We focus on CM energies above 500 GeV to avoid possible complications from large QCD corrections near the production threshold. In the energy region, an experimental target of top-quark physics is a precise measurement of the *Z*-top and top-Yukawa couplings. It is reasonable to expect that information beyond the standard theory could be probed through precise measurements of the top-production form factor [16]. To extract new physics from the form-factor measurement, one has to understand precisely the effects of higher-order corrections on the measurements. For instance, a signal of the scalar top in the MSSM can be observed through the loop effect in the top-quark production [36].

**Fig. 1:**
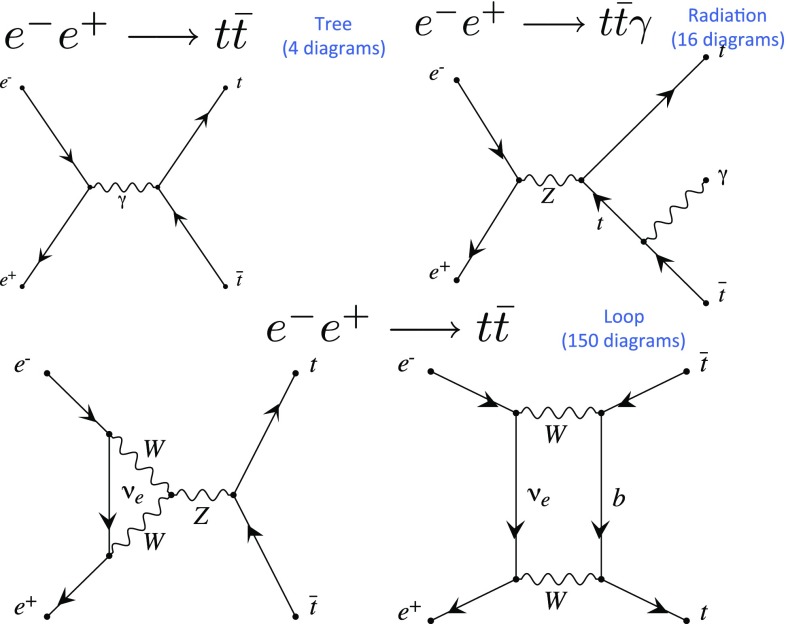
Examples of Feynman diagrams for $e^-e^+ \rightarrow t \bar{t}$ at tree level, with real radiation and at loop level. In our cross-section calculations, all diagrams include contributions from Goldstone bosons and light-fermion Yukawa couplings

For the $e^-e^+ \rightarrow t \bar{t}$ process, there are four Feynman diagrams at tree level, 16 with real-photon radiation, and 150 at the one-loop level. Typical diagrams are shown in Fig. 1.

**Fig. 2:**
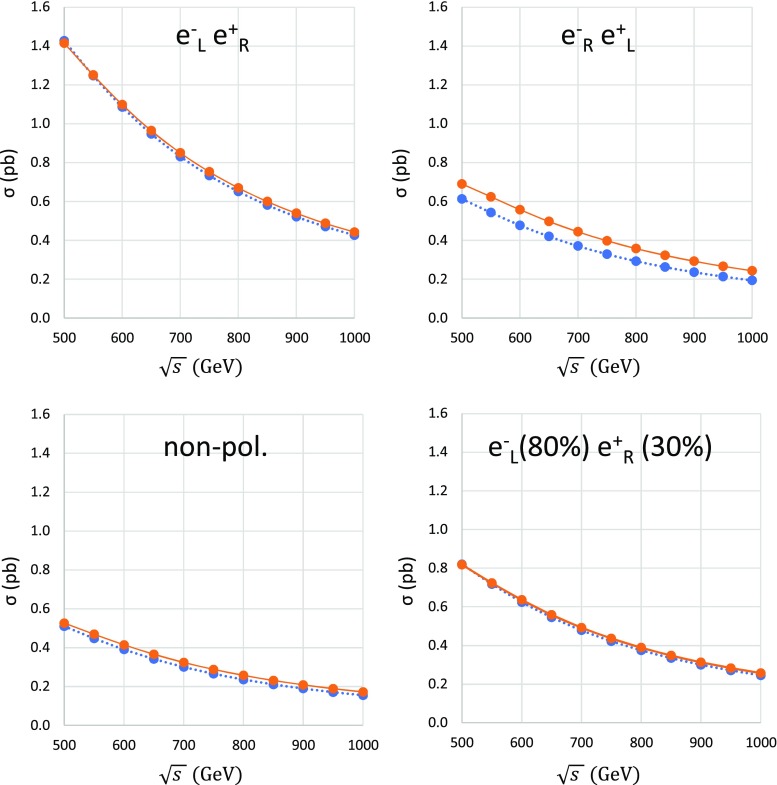
Total cross sections with respect to the CM energy $\sqrt{s}$ from 500 to 1000 GeV, assuming $100\%$ of $e^-_\mathrm{L}$ and $e^+_\mathrm{R}$ for the upper-left figure, and vice versa ($e^-_\mathrm{R}$ and $e^+_\mathrm{L}$) for the upper-right figure. Lower-left and lower-right figures show cross sections with non-polarization and polarization with a design value ($e^-_\mathrm{L}=80\%$ and $e^+_\mathrm{R}=30\%$, respectively). The dotted lines show the results for the tree level, while the solid lines correspond to the full one-loop electroweak correction

We calculate the total cross sections as a function of CM energy of 500–1000 GeV assuming $100\%$ left-hand polarization for electrons ($e^-_\mathrm{L}$) and $100\%$ right-hand polarization for positrons ($e^+_\mathrm{R}$), or vice versa ($e^-_\mathrm{R}$ and $e^+_\mathrm{L}$). The cross sections so obtained are shown in Fig. 2 as functions of the colliding energy. As shown in the upper panels of Fig. 2, the total cross sections for the $e^-_\mathrm{L}e^+_\mathrm{R}$ collision are roughly twice those for the $e^-_\mathrm{R}e^+_\mathrm{L}$ collision due to the *P*-violation of the weak interaction.

Cross sections with realistic polarizations of the design value ($e^-_\mathrm{L}=80\%$ and $e^+_\mathrm{R}=30\%$) can be obtained from those with $100\%$ polarized results as follows: the left-handed polarization degree of the electron beam is defined as $p_e=(N_\mathrm{L}-N_\mathrm{R})/(N_\mathrm{L}+N_\mathrm{R})$, where $N_\mathrm{L}$ and $N_\mathrm{R}$ are number of left-handed and right-handed electrons in the beam, respectively. When a normalization $N_\mathrm{L}+N_\mathrm{R}=1$ is used, the normalized number of left-handed and right-handed electrons can be obtained as $N_\mathrm{L}=(1+p_e)/2$ and $N_\mathrm{R}=(1-p_e)/2$, respectively. Therefore, the cross sections with left-handed electron polarization $p_e$ and right-handed positron polarization $p_p$ can be obtained as

$$\begin{aligned} \sigma (p_e,p_p)= & {} \frac{(1+p_e)(1+p_p)}{4}\sigma _{\mathrm{LR}}\\&+\frac{(1-p_e)(1-p_p)}{4}\sigma _{\mathrm{RL}}. \end{aligned}$$

where $\sigma _{\mathrm{LR}}$ ($\sigma _{\mathrm{RL}}$) are cross sections with the $100\%$ left polarized (right polarized) electron and the $100\%$ right polarized (left polarized) positron beams, respectively. We omit contributions involving $e^-_\mathrm{L} e^+_\mathrm{L}$ and $e^-_\mathrm{R} e^+_\mathrm{R}$ collisions because they yield negligible cross sections.

**Fig. 3:**
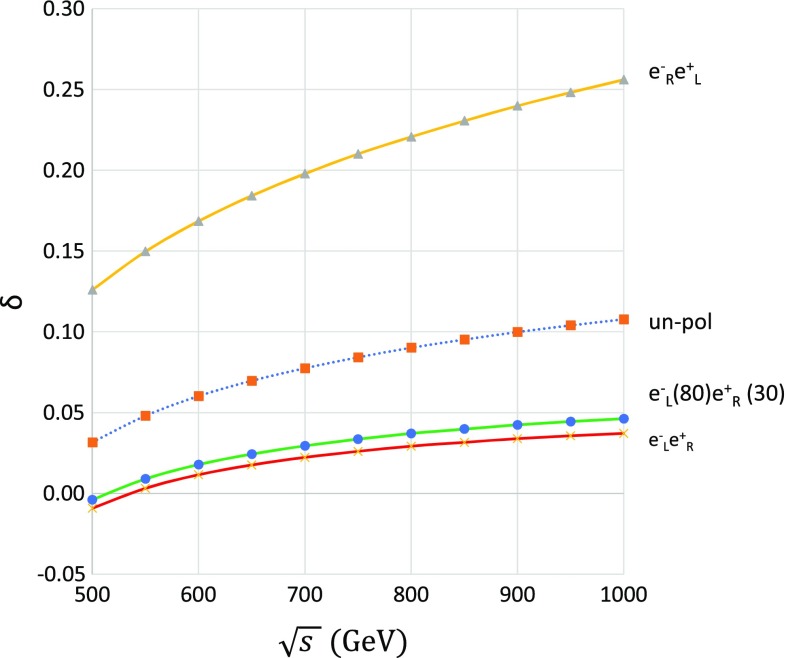
Ratio of the full correction $\delta$ for various polarization conditions. From the top of the figure, the lines show $e^-_\mathrm{R} e^+_\mathrm{L}$ polarization, non-polarization, design polarization, and $e^-_\mathrm{L} e^+_\mathrm{R}$ polarization, in that order

When design values of polarizations will be realized at the ILC, one can gain roughly $50\%$ in total cross section compared with the non-polarized case. In addition, the total amount of electroweak corrections is smaller for the $e^-_\mathrm{L}e^+_\mathrm{R}$ case than that for the $e^-_\mathrm{R}e^+_\mathrm{L}$ case. For a simple evaluation of the fraction of higher-order corrections, let us introduce the ratio $\delta =(\sigma _{\mathrm{NLO}}-\sigma _{\mathrm{Tree}})/\sigma _{\mathrm{Tree}}$, where $\sigma _{\mathrm{NLO}}$ and $\sigma _{\mathrm{Tree}}$ are the total cross sections at a full $\mathcal {O}(\alpha )$ correction and that at tree level, respectively. The results so obtained are summarized in Fig. 3. For instance, at a CM energy of 500 GeV, the electroweak correction of $e^-_\mathrm{L} e^+_\mathrm{R}$ is $-\,0.8\%$ and the electroweak correction of $e^-_\mathrm{R} e^+_\mathrm{L}$ is $12\%$. At a CM energy of 1000 GeV, the electroweak correction of $e^-_\mathrm{L} e^+_\mathrm{R}$ is $4.0\%$, where the electroweak correction of $e^-_\mathrm{R} e^+_\mathrm{L}$ is $26\%$. The $e^-_\mathrm{R} e^+_\mathrm{L}$ polarization has larger radiative corrections than those of the $e^-_\mathrm{L} e^+_\mathrm{R}$ one. Together with the larger cross sections, one can expect smaller systematic errors for the cross-section measurement with the polarized beam than in the non-polarized case. As shown in Fig. 3, an electroweak radiative correction gives very small radiative corrections on the polarized beam with the design value. While the non-polarized cross section also has small radiative corrections, the difference between the non-polarized and design polarized cases is significant. These small corrections on the total cross sections are due to the accidental cancellation among loop diagrams. This situation is suitable for new physics searches. If the top quark has anomalous couplings with gauge bosons, those signals can be observed with small systematic errors [16].

We note that the full electroweak correction reported here includes a trivial photonic correction from the initial-state photon radiation (ISR). It is well known that the ISR correction can be factorized and be improved using a higher-order re-summation [28]. The polarization asymmetry of electroweak corrections may be induced by diagrams involving *W* bosons [37], i.e., the diagrams shown in Fig. 1. In this report, we do not discuss the origin of the radiative-correction asymmetry in detail.

### Angular distributions

**Fig. 4:**
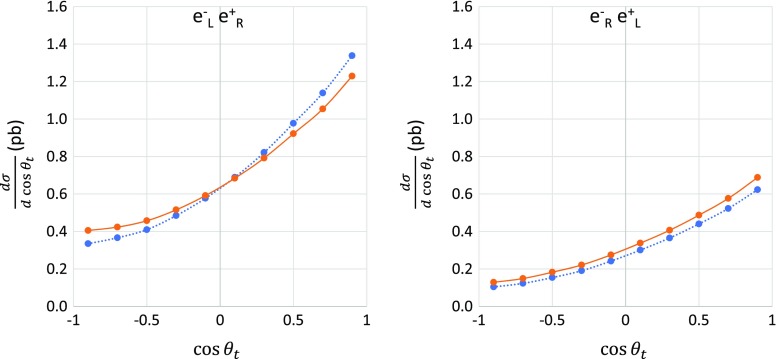
Angular distributions of the production angle of top quark $\theta _{top}$ at a CM energy of 500 GeV with $e^-_\mathrm{L} e^+_\mathrm{R}$ polarization (left) and $e^-_\mathrm{R} e^+_\mathrm{L}$ polarization (right). The dotted lines show tree-level results whereas the solid lines show full electroweak-corrected results

The angular distribution of the top-pair production has a large forward peak, and thus it has a sizable forward–backward asymmetry that allows us to make a good test of the standard theory. However, radiative corrections may distort the angular distribution as well as the total cross sections. Angular distributions of the top-pair production with and without radiative corrections at the CM energy of 500 GeV are shown in Fig. 4 for both $e^-_\mathrm{L} e^+_\mathrm{R}$ (left figure) and $e^-_\mathrm{R}e^+_\mathrm{L}$ (right figure) polarizations. The ISR corrections generally flatten the forward peak because of a smearing effect of the CM system. One can see this smearing effect clearly in the $e^-_\mathrm{L} e^+_\mathrm{R}$ polarization case. Even though the total correction $\delta$ is small at $\sqrt{s}=500$ GeV, as mentioned above, the electroweak correction modifies the angular distribution. A small correction to the total cross section is caused by an accidental cancellation between negative corrections for the forward region and a positive contribution in the backward region. In contrast, the electroweak correction for the $e^-_\mathrm{R} e^+_\mathrm{L}$ polarization gives positive corrections in the whole angular region, as shown in the right-hand panel in Fig. 4. In conclusion, the observed value of the forward–backward asymmetry is largely affected by the electroweak radiative corrections. Moreover, the effect of the radiative corrections depends on the spin polarization of the initial beams. Therefore, careful investigations of the forward–backward asymmetry are required.

A definition of the forward–backward asymmetry is given as follows. The forward and backward cross sections are defined as $\sigma _\mathrm{F}= \int _{0}^{1} {\mathrm{d}\sigma } / {\mathrm{d}\cos {\theta _t}} ~\mathrm{d}\cos {\theta _t}$ and $\sigma _\mathrm{B}= \int _{-1}^{~0} {\mathrm{d}\sigma } /{\mathrm{d}\cos {\theta _t}} ~\mathrm{d}\cos {\theta _t}$, respectively. Thus, the forward–backward asymmetry is defined by $A_{\mathrm{FB}}= ( {\sigma _\mathrm{F}-\sigma _\mathrm{B}})/ ( \sigma _\mathrm{F}+\sigma _\mathrm{B})$. The tree and electroweak-corrected values of the forward–backward asymmetry at the CM energy of 500 GeV are summarized in Table 2. For $e^-_\mathrm{L} e^+_\mathrm{R}$ ($e^-_\mathrm{R} e^+_\mathrm{L}$) polarization, the forward–backward asymmetry at tree level is 0.385 (0.467), which becomes 0.317 (0.443) after the full electroweak correction. When the design values of polarizations are assumed, the forward–backward asymmetry is determined mainly by the contribution from the $e^-_\mathrm{L} e^+_\mathrm{R}$ component, as shown in the last row of Table 2.

### Top-quark decay

**Fig. 5:**
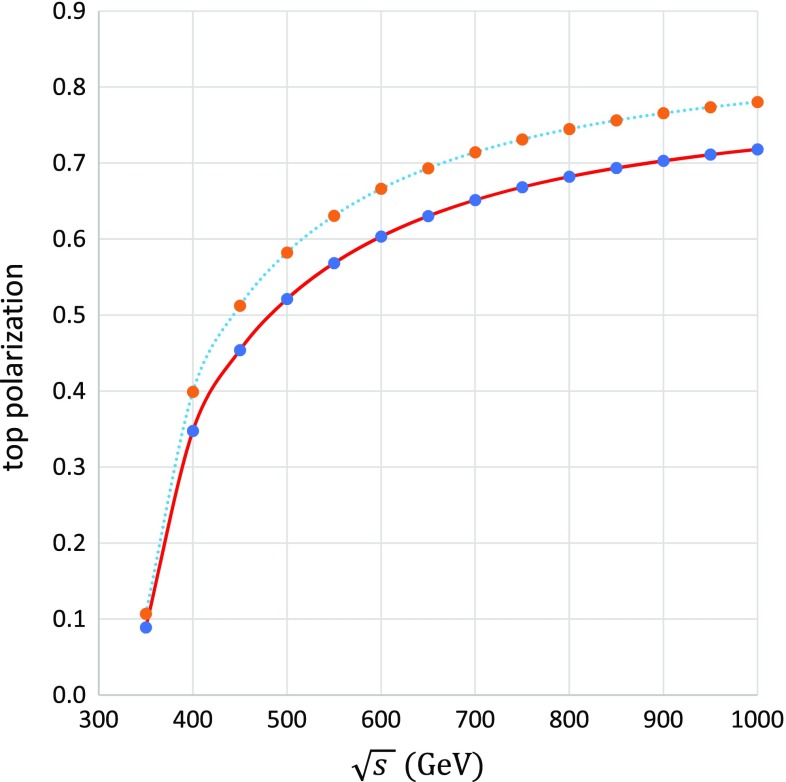
Top-quark polarization as a function of the CM energy from 300 to 800 GeV for the process $e^-_\mathrm{L} e^+_\mathrm{R} \rightarrow t \bar{t}$. The dotted lines show tree-level results, whereas the solid lines show full electroweak-corrected results

According to the beam polarization, the produced top quarks are also polarized. The polarization degree is defined as $\delta _{pol}=({\sigma _\mathrm{L} - \sigma _\mathrm{R}})/({\sigma _\mathrm{L} + \sigma _\mathrm{R}})$, where $\sigma _\mathrm{L}$ and $\sigma _\mathrm{R}$ are the cross sections for creating the left-handed and right-handed top quark, respectively. The polarization degree depends on the CM energy, as shown in Fig. 5. At tree level, the polarization degree increases from $8.8\%$ at 350 GeV to 67.6% at 800 GeV. At a CM energy of 350 GeV (close to the production threshold), the produced top quark moves slowly and thus its helicity state is easily flipped. In contrast, at higher energies, the particle moves much faster and the helicity is stable. That causes the difference in polarization to increase with energy, as shown in Fig. 5. The full electroweak corrections reduce the polarization degree by roughly $10\%$ in the high-energy region. The top quark immediately decays into a bottom quark and a fermion pair. Because the angular and energy distributions of the decay products depend strongly on the top polarization, an exact treatment of the top polarization is mandatory. We discuss the top decay of $t \longrightarrow b\mu ^+\nu _{\mu }$ at a CM energy of 500 GeV as a benchmark process. Because b-quark tagging is required to identify the top quark experimentally, precise calculation of b-quark distributions is important.

**Table 2:** Estimated values of the forward–backward asymmetry at a CM energy of 500 GeV

$e^-e^+\rightarrow t\bar{t}$	$A_{\mathrm{FB}}$ (Tree)	$A_{\mathrm{FB}}$ (Full)
$e^-e^+$	0.410	0.359
$e^-_\mathrm{L}e^+_\mathrm{R}$	0.385	0.317
$e^-_\mathrm{R}e^+_\mathrm{L}$	0.467	0.443
$e^-_\mathrm{L}(80\%)e^+_\mathrm{R}(30\%)$	0.388	0.321

The number of Feynman diagrams for the six-body final state $e^- e^+ \rightarrow b \bar{b}\mu ^-\mu ^+\nu \bar{\nu }$ is too large, and thus a full electroweak correction is impossible using the current computing power. Instead, we have used a narrow-width approximation (NWA) for the top-quark production and decay, including the spin correlation exactly. A more sophisticate method to treat a particle production and decay consistently at a one loop order is known as the double-pole approximation. This method is developed for a *W*-boson pair production [38, 39] at first, and later it is applied to a top-quark production [40] too. We do not employ the double-pole approximation in this study, because a simple NWA is enough to discuss an effect of electroweak corrections on a top-quark polarization. E.g., an energy distribution of decayed b-quarks is mainly determined by the top-quark polarization degree.

The branching ratio of the $b\mu ^+\nu _{\mu }$ decay is obtained with the $\mathcal {O} (\alpha )$ correction as follows: the top width at tree level is calculated to be $\Gamma ^{\mathrm{Tree}}=1.416$ GeV. The full electroweak-corrected width is calculated by summing all possible decay channels of $t\rightarrow b l \nu _l$ and $t\rightarrow b q \bar{q}$ as $\Gamma ^{\mathrm{Loop}}=1.421$ GeV. The partial width of the decay channel to $b\mu ^+\nu _{\mu }$ is 0.1535 GeV, thus the branching ratio of this channel is obtained as $10.8\%$ after the $\mathcal {O}(\alpha )$ correction. Here, only electroweak corrections are included. The effect of the QCD higher-order correction is known to be about $-\,5\%$ (hadronic decays) and $-\,9\%$ (semi leptonic decays) [20, 41], and they are not included in this study. In our approximation, corrections on the top-quark width affect only on the branching ratio of some specific decay channel, and then they does not affect on any distributions. On the other hand, radiative corrections on the top-quark spin polarization largely affect on energy distributions of b-quarks.

**Fig. 6:**
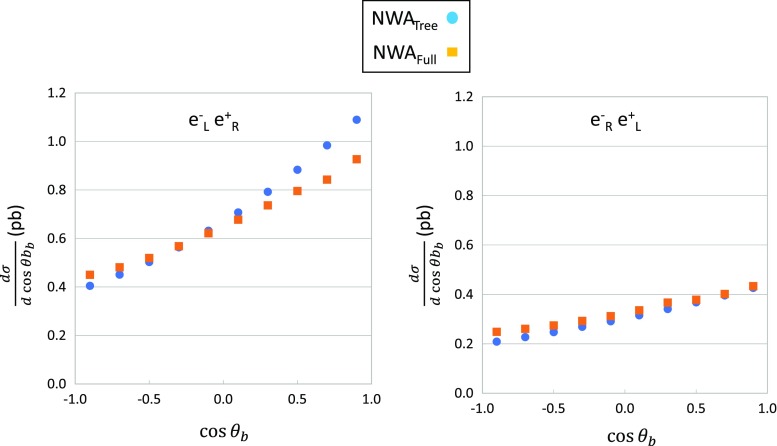
Angular distributions of b-quarks with $e^-_\mathrm{L} e^+_\mathrm{R}$ (left) and $e^-_\mathrm{R}e^+_\mathrm{L}$ (right) polarizations. Circle and square points show tree and electroweak-corrected distributions, respectively

The total cross section of *N*-body production including a narrow fermion resonance with mass *m* and width $\Gamma$, which decays into *N* bodies, can be expressed as

$$\begin{aligned} \sigma= & {} \frac{1}{flux} \int ~ {\left| \mathcal {M} \right| }^2 \mathrm{d}\Omega _N\\= & {} \frac{1}{flux} \int \frac{{\left| \sum _\lambda \mathcal {M}_p u_{\lambda }(q)\bar{u}_{\lambda }(q)\mathcal {M}_d \right| }^2}{(q^2-m^2)^2+m^2\Gamma ^2}\\&\times \, \frac{dq^2}{2\pi } \mathrm{d}\cos {\theta _q} \mathrm{d}\varphi _q \mathrm{d}\Omega _n \mathrm{d}\Omega _{N-n}, \end{aligned}$$

where $u_{\lambda }$ is the spinor, $q_{\mu }$ is the momentum (off-shell), and $\lambda$ is the spin of the resonance particle. The term $\mathrm{d}\Omega _n$ denotes an *n*-body phase space, and $\mathcal {M}_p$ and $\mathcal {M}_d$ are the product and decay amplitudes, respectively. Using an on-shell approximation as $q^2\sim q_0^2=m^2$ for the numerator, the amplitudes can be approximated by $\tilde{\mathcal {M}}_p^{\lambda }=\mathcal {M}_p u_{\lambda }(q_0)$ and $\tilde{\mathcal {M}}_d^{\lambda }=\mathcal {M}_d u_{\lambda }(q_0)$. Therefore, the total cross section becomes

$$\begin{aligned} \sigma\simeq & {} \frac{1}{flux} \sum _{\lambda } \int {\left| \tilde{\mathcal {M}}_p^{\lambda } \right| }^2 \mathrm{d}\cos {\theta _q} \mathrm{d}\varphi _q \mathrm{d}\Omega _{N-n}\\&\int {\left| \tilde{\mathcal {M}}_d^{\lambda } \right| }^2 \mathrm{d}\Omega _n \int \frac{1}{(q^2-m^2)^2+m^2\Gamma ^2} \frac{dq^2}{2\pi }. \end{aligned}$$

We note that the spin correlation is maintained between production and decay. Integration can be performed over the resonance masses, namely

$$\begin{aligned}&\int {\left| \tilde{\mathcal {M}}_d^{\lambda } \right| }^2 \mathrm{d}\Omega _n \int _{-\infty }^{+\infty }~ \frac{1}{(q^2-m^2)^2+m^2\Gamma ^2} \frac{dq^2}{2\pi }\\&\quad = \frac{1}{\Gamma } \frac{1}{2m} \int {\left| \tilde{\mathcal {M}}_d^{\lambda } \right| }^2 \mathrm{d}\Omega _n, \end{aligned}$$

which gives the branching ratio of a specific decay channel. In reality, calculations are performed using the exact six-body phase space. The validity of the NWA is verified by comparing b-quark distributions obtained by the narrow-width and the exact six-body calculations at tree level. Both results agree each other within the statistical error of Monte Carlo calculations. Since the contribution from non-resonant diagrams is negligible [17] up to the CM energies considering in this study, the NWA are precise enough. For a higher energy region than at TeV order, the contribution from non-resonant diagrams becomes important [18].

**Fig. 7:**
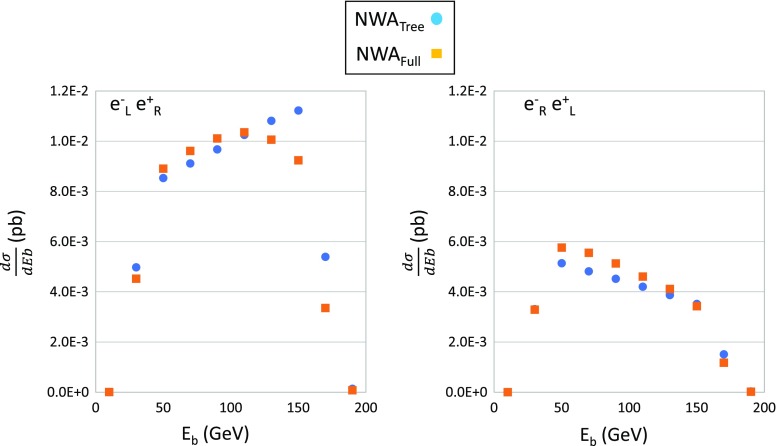
Energy distributions of b-quarks with $e^-_\mathrm{L} e^+_\mathrm{R}$ (left) and $e^-_\mathrm{R}e^+_\mathrm{L}$ (right) polarizations. Circle and square points show tree and electroweak-corrected distributions, respectively

The angular and energy distributions of b-quarks are shown in Figs. 6 and 7, respectively. For the $e^-_\mathrm{L} e^+_\mathrm{R}$ polarization case, the decayed b-quarks tend to be produced in the forward direction of the top-quark momentum, and in the backward direction for the $e^-_\mathrm{R} e^+_\mathrm{L}$ polarization. The angular distributions of the b-quarks at tree level reflect this tendency. The electroweak corrections distort the angular distribution rather largely in the $e^-_\mathrm{L} e^+_\mathrm{R}$ polarization case, as shown in the left-hand panel of Fig. 6.

In the top-quark rest frame, the b-quark energy is monochromatic (while ignoring the *W*-boson width). Thus, the energy distribution of the b-quarks are a reflection of their angular distribution with respect to the top-quark momentum, after the Lorentz boost due to finite top-momentum. From this point on view, the energy distribution of b-quarks can be understood intuitively. Again, the electroweak corrections distort the distribution largely for the $e^-_\mathrm{L} e^+_\mathrm{R}$ case, as shown in Fig. 7.

While these effects on the decay products from the higher-order corrections are important for the precise estimation of the event acceptance, it is also important for the new physics searches. For instance, it is reported that the spin correlation between top and anti-top quarks is sensitive to the BSM [42]. The spin polarization of (anti-)top quarks is affected by the electroweak radiative correction, it is important to include effects from the radiative corrections in such kind of analysis in the ILC experiments.

### QCD correction

**Fig. 8:**
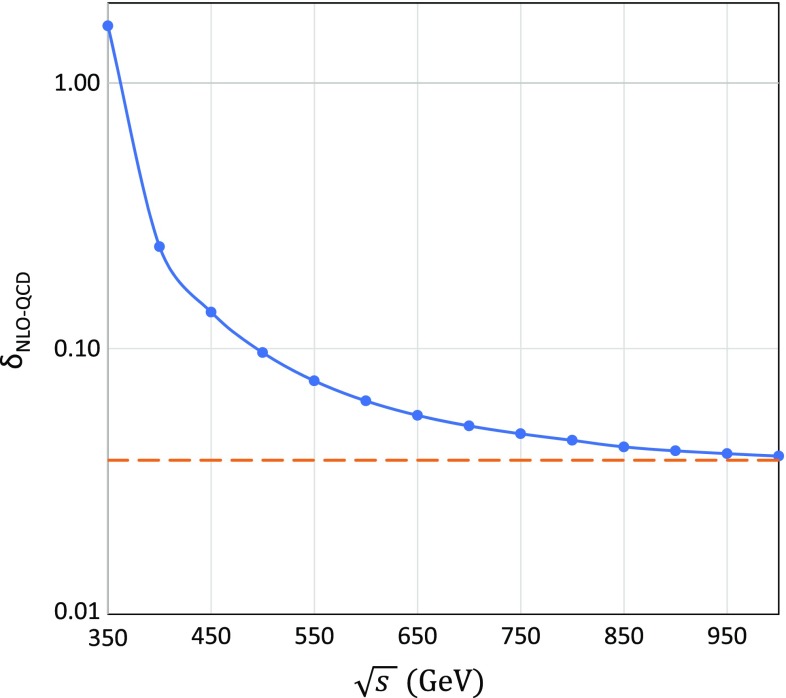
NLO–QCD correction of the top-pair production process. A strong coupling constant $\alpha _s=0.12$ is used

We have not discussed the QCD correction so far in this report because the QCD correction for the top-pair production is independent of the beam polarization and simply modifies the total cross section while maintaining the distributions. However, the QCD correction is not small at a CM energy of 500 GeV. The formulas used here are summarized in Appendix A. While the QCD correction is expected to be $\alpha _s/\pi \simeq 3.8\%$ at higher energies, it still makes a contribution of $9.7\%$ to the total cross section at a CM energy of 500 GeV. While the QCD correction gradually approaches the asymptotic value of $\alpha _s/\pi$ with increase of the CM energy, as shown in Fig. 8, it still makes a large contribution around a CM energy of 500 GeV. While results including only electroweak corrections are shown in this report, more precise QCD corrections [18, 43] must be included for future experimental analysis.

## Summary and conclusions

In this report, we have presented full $\mathcal {O}(\alpha )$ electroweak corrections for the $e^-e^+\rightarrow t \bar{t}$ process associated with the sequential decay $t\rightarrow b \mu \nu _\mu$. Calculations were performed using the GRACE-Loop system. The electroweak radiative correction was estimated typically at a level of $10\%$ on the total cross section in the on-shell scheme for the non-polarized case. While the cross section with $e^-_\mathrm{L} e^+_\mathrm{R}$ polarization was roughly twice that with $e^-_\mathrm{R} e^+_\mathrm{L}$ polarization at tree level, the radiative correction of the former was smaller than that of the latter. The electroweak correction with the design polarizations ($e^-_\mathrm{L}=80\%$ and $e^+_\mathrm{R}=30\%$) was estimated to be less than $5\%.$ Even though the electroweak correction of the total cross sections was rather small for $e^-_\mathrm{L} e^+_\mathrm{R}$ polarization, the radiative corrections modified the angular distribution of the produced top quarks. The radiative corrections decreased the forward–backward asymmetry of the top-quark production from 0.388 to 0.321 for the design polarization. We also studied the properties of top-quark decay $t \rightarrow b\mu ^+\nu _{\mu }$ including the spin correlation. Both production and decay processes were calculated with $\mathcal {O} (\alpha )$ corrections and combined with using the narrow-width approximation. We observed the energy distribution of b-quarks to be largely distorted because of the radiative correction. Therefore, an event generator including radiative corrections for both production and decay with the spin correlation will be necessary for precise measurements in future ILC experiments. Because the NLO–QCD correction is still large at CM energies of 500 GeV, a precise QCD correction is also desired.

The authors wish to thank Prof. J. Vermaseren and Prof. J. Fujimoto for their continuous encouragement and fruitful discussions. T.U. is supported by the ERC Advanced Grant No.320651 “HEPGAME”.

## Appendix A: QCD correction

The detailed formulas of the NLO–QCD correction for massive quark-pair production by electroweak interaction are summarized in this appendix. In the following calculations, the standard $\overline{\mathrm {MS}}$ renormalization scheme is used. After renormalization, a space-time dimension other than four is reinterpreted to regulate the infrared divergence as $d=4-2\varepsilon _{\mathrm{UV}}\rightarrow 4+2\varepsilon _{\mathrm{IR}}$ with $\varepsilon _{\mathrm{IR}}>0$. The NLO–QCD correction consists of three parts: vertex, self-energy, and real-gluon-emission corrections. The contributions of each part are given separately below.

### Vertex correction

The total vertex correction is given as

$$\begin{aligned} \Gamma= & {} C_\mathrm{F}\left( I_k+I_0\right) , \end{aligned}$$

where $C_{F}=4/3$ is a color factor. Each integration term is given as

$$\begin{aligned} I_k= & {} \frac{\alpha _s}{4\pi }\left\{ \frac{-1}{\varepsilon _{\mathrm{IR}}}+\left( L^{\prime }{}+\log {\mu _t}+1+\tilde{\mu }_t\log {\left( -\frac{1-\tilde{\mu }_t}{1+\tilde{\mu }_t}\right) } \right) \right\} ,\\ I_0= & {} \frac{\alpha _s}{4\pi }\left\{ \frac{1}{\varepsilon _{\mathrm{IR}}}\frac{2\left( 2\mu _t+1\right) }{\tilde{\mu }_t} \log {\left( -\frac{1-\tilde{\mu }_t}{1+\tilde{\mu }_t}\right) }\right. \\&-\,1-\frac{2\left( 2\mu _t+1\right) }{\tilde{\mu }_t} \left( Sp\left( \frac{1}{2}-\frac{1}{2\tilde{\mu }_t}\right) - Sp\left( \frac{1}{2}+\frac{1}{2\tilde{\mu }_t}\right) \right) \\&+\,\frac{2}{\tilde{\mu }_t}\log {\left( -\frac{1-\tilde{\mu }_t}{1+\tilde{\mu }_t}\right) }\left( -2\left( 6\mu _t+1\right) \right. \\&+\,\left( 2\mu _t+1\right) \left[ L^{\prime }{}+\frac{1}{2}\log {\left( -\frac{1-\tilde{\mu }_t}{1+\tilde{\mu }_t}\right) }\right. \\&\left. \left. \left. + \log {\left( -\frac{\tilde{\mu }_t(\tilde{\mu }_t+1)}{2}\right) } \right] \right) \right\} ,\\ L^{\prime }{}= & {} \log {\left( \frac{-s}{\mu _\mathrm{F}}\right) },~~\mu _t=-\frac{m_t^2}{s},~~\tilde{\mu }_t=\sqrt{4\mu _t+1}, \end{aligned}$$

where $\mu _\mathrm{F}$ is the factorization energy scale, $m_t$ is the top-quark mass, and *s* is the momentum square of a $t\bar{t}$-system.

### Self-energy correction

The self-energy correction appears because of the renormalization scheme. The top mass that appears here must be interpreted as the $\overline{\mathrm {MS}}$ mass:

$$\begin{aligned} \Sigma \left( p^2=m_t^2\right)= & {} C_\mathrm{F}\frac{\alpha _s}{4\pi }\left\{ \frac{-1}{\varepsilon _{\mathrm{IR}}}+\left( L_t-4\right) \right\} , \end{aligned}$$

where $$L_t=\log {\left( {m_t^2}/{\mu _\mathrm{F}^2}\right) }$$.

### Real-emission correction

The real-emission correction is further separated into two parts: soft-gluon emission and hard-gluon emission. A threshold energy $k_c$ is introduced to separate soft and hard emissions. The soft-emission corrections are given as

$$\begin{aligned} R_{ii}= & {} C_\mathrm{F}\frac{\alpha _s}{2\pi }\left\{ \frac{1}{\varepsilon _{\mathrm{IR}}}-L_k-\frac{1}{\tilde{\mu }_t} \log {\left( -\frac{1-\tilde{\mu }_t}{1+\tilde{\mu }_t}\right) } \right\} ,\\ R_{ij}= & {} C_\mathrm{F}\frac{\alpha _s}{2\pi }\left\{ \frac{-1}{\varepsilon _{\mathrm{IR}}}\left( \frac{2\mu _t+1}{\tilde{\mu }_t}\right) \log {\left( -\frac{1-\tilde{\mu }_t}{1+\tilde{\mu }_t}\right) }\right. \\&-\,\frac{2\mu _t+1}{\tilde{\mu }_t}\left. \left( L_k\log {\left( -\frac{1-\tilde{\mu }_t}{1+\tilde{\mu }_t}\right) }\right. \right. \\&\left. \left. +\,Sp\left( \frac{2}{1+1/\tilde{\mu }_t}\right) - Sp\left( \frac{2}{1-1/\tilde{\mu }_t}\right) \right) \right\} , \end{aligned}$$

where $L_k=2\log {(2k_c/\mu _\mathrm{F})}$. These formulas are obtained via an approximation in which the gluon energy is much smaller than $m_t$. The hard-emission cross section can be calculated using the GRACE system based on the exact matrix element.

We confirmed numerically that real-emission corrections are independent of $k_c$, whose values are below 1 GeV.

### Total correction

The NLO–QCD cross section $\sigma _{\mathrm{NLO}}$ can be obtained as

$$\begin{aligned} \sigma _{\mathrm{NLO}}= & {} \left\{ 1+2\left( R_{ii}+R_{ij}+\mathrm {Re}\left[ \Gamma +\Sigma \right] \right) \right\} \sigma _0+\sigma _g, \end{aligned}$$

where $\sigma _0$ and $\sigma _g$ are the Born and hard-emission cross sections, respectively. After summing up all contributions, the infrared divergence and $\mu _\mathrm{F}$ dependence disappear completely.

## Footnotes

1 For the most up-to-date results, see [4] for ATLAS and [5] for CMS collaborations.
